# ACE Inhibitory Peptides Derived from Muscovy Duck (*Cairina moschata*) Plasma

**DOI:** 10.3390/foods12010050

**Published:** 2022-12-22

**Authors:** Zongshuai Zhu, Haoyu Guo, Yan Xu, Anthony Pius Bassey, Ahtisham Ali, Ming Huang, Jichao Huang

**Affiliations:** 1 College of Food Science and Technology, Nanjing Agricultural University, Nanjing 210095, China; 2 College of Engineering, Nanjing Agricultural University, Nanjing 210031, China

**Keywords:** Muscovy duck plasma, ACE inhibitory peptide, purification, identification, molecule docking

## Abstract

In this study, angiotensin-converting enzyme inhibitory peptides (ACE–IPs) derived from Muscovy duck (*Cairina moschata*) plasma hydrolysate (MDPH) were investigated. According to the general research protocol for bioactive peptides, the crude ACE–IPs of Muscovy duck plasma were separated and purified by ultrafiltration, gel chromatography and reversed-phase high-performance liquid chromatography (RP–HPLC). Then the components with the highest ACE inhibition potential were selected for identification. Finally, the inhibition mechanism was explored by molecular docking and in silico simulated digestion. A total of 121 peptides was detected, and five were screened for synthesis verification and molecular docking. The peptide VALSSLRP revealed high ACE inhibitory activity (91.67 ± 0.73%) because this peptide bound tightly to the S1′ pocket and formed 3 hydrogen bonds. Meaningfully, this work provides some new information about the generation of ACE–IPs derived from duck blood plasma.

## 1. Introduction

Muscovy duck (*Cairina moschata*), introduced from abroad to mainland China, is a special type of waterfowl that belongs to different species from ordinary domestic ducks [1,2]. Due to its high meat yield, high heat resistance and good resistance to adverse environments and diseases, Muscovy duck has become a meat duck species and is developing rapidly in China among meat consumers [3]. Duck blood is an integral byproduct from duck meat processing. Even though duck blood is an abundant source of inexpensive proteins, its utilization is limited. Following its dark red color and the strong metallic taste of blood, duck blood is directly discarded into the environment without proper disposal, which limits further application as a food ingredient for direct human consumption and causes resources to be wasted and the environment polluted [4]. Hence, converting the waste into high value-added products is necessary to contribute to its economic utilization.

Some researchers have investigated that specific peptide from animal blood possesses bioactivity, such as antioxidants. For instance, Yang et al. (2020) [4] demonstrated that the alcalase hydrolysate from duck plasma powder exhibited strong DPPH radical-scavenging activity. Zheng et al. (2018) [5] prepared antioxidant peptides and optimized the hydrolysis conditions from chicken blood cells with papain and flavorzyme and found that AEDKKLIQ (943.5 Da) exhibited free radicals-scavenging potential and reducing power as well as that of glutathione. However, ACE–IPs derived from poultry blood were rarely reported.

ACE inhibition is one of the fundamental approaches to treating hypertension and associated diseases through maintaining angiotensin I, angiotensin II or aldosterone level to balance the renin–angiotensin system (RAS) and kallikrein–kinin system (KKS) [6]. However, long-term application of synthetic ACE inhibitors, including enalapril, lisinopril, captopril, etc., will induce side effects like nausea, headache, cough and severe cases like proteinuria and blood dyscrasias [7,8]. Therefore, naturally-derived bioactive peptides with ACE inhibition potential were considered to eliminate the side effects and be safer than pharmaceutical drugs, leading to the focus on natural ACE inhibitors [9,10].

It has been reported that ACE–IPs were isolated from beans, cereal, vegetables, meat, milk and eggs [8,11,12,13,14,15]. Recently, ACE–IPs were found also in protein extracts from insects (*Tenebrio molitor*), in yeast extracts and in spirulina (*Arthrospira platensis*) [16,17,18]. But there is little or no information on Muscovy duck plasma. Therefore, Muscovy duck plasma hydrolysate with ACE inhibitory potential was prepared using enzymatic hydrolysis.

This study aimed to isolate and identify novel ACE–IPs and explore the interactions between purified peptides and ACE. To achieve this objective, the plasma’s crude peptides were successively separated and purified by ultrafiltration, gel filtration chromatography and reversed-phase high-performance liquid chromatography (RP–HPLC). The samples were identified using Nano-LC–ESI–MS/MS to obtain the amino acid (AA) sequences of the peptides. After screening, the selected peptides were synthesized, and their ACE inhibitory activity was verified. Additionally, molecular docking and in silico simulated peptide digestion were employed to reveal the molecular interactions between single peptides and ACE. Considering the synthetic peptides’ activity, molecular docking and in silico simulated peptide digestion results, the ACE inhibitory mechanism of peptides was also analyzed. The production and utilization of ACE–IPs from Muscovy duck plasma (MDP) could reduce economic costs and environmental conservation.

## 2. Materials and Methods

### 2.1. Materials

Muscovy ducks (*Cairina moschata*) blood was obtained from Anqing Yongqiang Agricultural Science and Technology Co., Ltd. (Anqing, China). Alkaline protease (S10154, 200 U/mg) was purchased from Yuanye Biotechnology (Shanghai, China). Bicinchoninic acid (BCA) protein assay kit (Biosharp, Shanghai, China) was employed for protein content determination. ACE from rabbit lung (0.1 U/mL) and hippuryl–L–histidyl–L–leucine (HHL) was purchased from Sigma-Aldrich (St. Louis, MO, USA). Acetonitrile, formic acid and triethylamine were HPLC grade, while other reagents were of analytical grade.

### 2.2. MDPH Preparation

Muscovy duck blood was collected in sterilized glass bottles that contained sodium citrate solution as an anticoagulant (final concentration was 1%, *w*/*v*). The collected blood was transported to the laboratory under 4°C. The whole blood was immediately centrifuged (10,000× *g*, 4°C, 10 min), which was separated into MDP (protein concentration was determined to be 40.11 ± 1.31 mg/mL) and blood cells.

MDP was adjusted to pH 10.5 with 1 M NaOH and pre-incubated (65.5°C, 10 min) in a water bath, then hydrolyzed by alkaline protease (2500 U/g) at 65°C for 5.85 h. During the enzymatic hydrolysis, the solution pH was adjusted to 10.5 every 30 min. After the reaction, the pH was adjusted to 7 with 1 M HCl, and alkaline protease was inactivated for 10 min using a boiling water bath. Then the solution was rapidly cooled in an ice bath and centrifuged (10,000× *g*, 10 min, 4°C). The supernatant (termed “MDPH”) was stored at 4°C for further analysis.

### 2.3. ACE Inhibition Activity

The method of Shi et al. was employed to measure the ACE inhibition activity [14]. ACE, HHL and samples were dissolved in 100 mM borate buffer containing 300 mM NaCl at pH 8.3. Firstly, an aliquot of 200 µL of the substrate (HHL, 5 mM) was mixed with 80 µL of samples (1 mg/mL) or borate buffer solution, and the mixture was pre-incubated (7°C, 10 min) in a 2 mL polypropylene centrifuge tube. Then, an aliquot was incubated with 20 µL of ACE (0.1 U/mL) at 37°C for 1 h. The reaction was stopped by adding 150 µL of 1 M HCl solution. The obtained solution was filtered through a 0.22 µm water filtration membrane and analyzed on an HPLC system (Acquity H-Class, Waters co., Huntington Beach, CA, USA) with a Sunfire C18 analytical column (4.6 mm × 150 mm, particle size 5 µm, Waters Scientific Inc., Milford, MA, USA). Mobile phases were: (A) ultra-pure water containing 0.1% formic acid and triethylamine; (B) acetonitrile containing 0.1% formic acid and triethylamine. The sample was eluted by an isocratic elution of 85% A and 15% B at a flow rate of 1 mL/min. The sample and column temperature were set at 30°C, and the injection volume was 10 µL. Absorbance was monitored at 228 nm. The activity was calculated as follows:

$$\begin{array}{c} \begin{array}{c} ACE\;inhibitory\;activity\;\left(\%\right)\;=\frac{A_{\mathrm{blank}}-A_{\mathrm{sample}}}{A_{\mathrm{blank}}}\;\times \;100\% \end{array} \end{array}$$

where A_blank_ and A_sample_ represent the peak areas of the chromatographic peak of hippuric acid in the blank (borate buffer) and the test sample, respectively.

### 2.4. Peptide Separation and Purification

#### 2.4.1. Ultrafiltration

MDPH was ultrafiltered by a 3 kDa molecular weight (MW) cut-off (MWCO) membrane (Millipore, Burlington, MA, USA). Then two MDPH fractions with UF-I (<3 kDa) and UF-II (>3 kDa) were collected. Each fraction was freeze-dried and stored at −20 °C. Then, these two fractions were tested for ACE inhibitory activity to select the one showing higher activity for further purification and analysis.

#### 2.4.2. Gel Filtration Chromatography

The procedure of Li et al. was explored with minor changes [19]. The fraction exhibiting the highest ACE inhibitory activity was purified via gel filtration chromatography with the Sephadex G-25. The sample (2 mL, 60 mg/mL) was loaded onto the column (1.6 × 70 cm), using ultra-pure water as the elution buffer at a 2 mL/min flow rate. The absorbance of the solutions was measured at 280 nm. The fractions were collected using an automatic fraction collector and freeze-dried to test ACE inhibitory activity and stored at −20°C for further purification.

#### 2.4.3. RP–HPLC

The procedure outlined by Cao et al., was employed with minor adjustments [20]. The most active fraction was further purified by RP–HPLC on a semi-preparative C18 column (5 µm, 10.0 × 250 mm) on Agilent 1200 HPLC system (Agilent Technologies, Santa Clara, CA, USA) equipped with a photodiode array detector. The sample (100 µL, 20 mg/mL) was eluted with a linear gradient at the flow rate of 0.6 mL/min. Mobile phases were: (A) ultra-pure water containing 0.1% formic acid; (B) acetonitrile containing 0.1% formic acid. The elution was performed in the following sequence: 0–25 min, 2–20% B; 25–35 min, 100% B; 35–40 min, 20–2% B. Peaks detected at 214 nm were collected for testing ACE inhibitory activity and stored at −20°C for identification.

### 2.5. Identification of Peptide Sequences by LC–MS/MS

The AA sequences of the fraction exhibiting the strongest inhibition potential were identified by HPLC (Thermo Fisher, Waltham, MA, USA) coupled with the Q-Exactive mass spectrometer (Thermo Fisher, Waltham, MA, USA). The purified peptide fraction was applied to a liquid chromatographic column (0.15 × 150 mm, Column Technology Inc., Fremont, CA, USA). Mobile phases were: (A) ultra-pure water containing 0.1% formic acid; (B) 84% acetonitrile containing 0.1% formic acid. Elution condition was: 0–50 min, B 4–50%; 50–54 min, B 50–100%; 54–60 min, B 100%. The loading volume was 5 μL, and the flow rate was 0.3 mL/min. The capillary temperature and ionization voltage were set to be 200°C and 2.2 kV, respectively. Afterwards, mass spectrometry (MS) was performed using a Q-Exactive mass spectrometer. The data was acquired using a data-dependent top10 method dynamically choosing the most abundant precursor ions from the survey scan (300–1800 *m*/*z*) for HCD fragmentation. The test conditions were as follows: Spray voltage positive ion 2000 V, ion transfer tube temperature 275°C, S-Lens RF level 60, scan range 350–1800 *m*/*z*, orbitrap resolution 60 K, collision energy 32%, isolation window 1.6, analysis duration 60 min. MS test raw file (Raw File) with software Mascot 2.2 was used to search the corresponding database (Mascot Databases: accessed on 1 December 2022, https://hpc.nih.gov/docs/mascot_databases.php) to obtain the protein results.

### 2.6. Prediction of ACE Inhibitory Activity

The sequence of peptides identified in the MDPH was further screened for ACE–IPs through the electronic prediction of antihypertensive peptides on the AHTPin online website (accessed on 1 December 2022, http://crdd.osdd.net/raghava/ahtpin).

### 2.7. Peptide Synthesis

The purified peptide was synthesized using standard Fmoc-chemistry at China Peptides Co., Ltd. (Wuhan, China), with purities >98%.

### 2.8. Molecular Docking

The ACE crystal structure (PDB ID: 1O86) was derived from RCSB Protein Data Bank (accessed on 1 December 2022, http://www.rcsb.org), and the 3D structures of the identified peptides were prepared by UCSF Chimera 1.16 (University of California, San Francisco, CA, USA). The AutoDock Tool was used to add hydrogens, remove water, transform the files from .pdb format to pdbqt format and determine the target’s docking box. Molecular docking between ACE–IPs and ACE (PDB ID: 1O86) was performed using AutoDock Vina. The best binding mode was chosen based on the lowest binding energy. Pymol software was used to analyze the interactions between ACE and the peptides.

### 2.9. In Vitro Simulated Pepsin and Trypsin Digestion

MDPH *in vitro* simulated pepsin and trypsin digestion was determined as outlined by Shi et al. [14] with minor adjustments. The activity of two enzymes involved in gastrointestinal digestion was tested. The MDPH was incubated with 4% pepsin (16,000 U/g) at pH 2 and 37°C for 2 h. After the reaction, the pH was adjusted to 5.3 using 0.9 M NaHCO_3_ and readjusted to 7 using 4 M NaOH. Pepsin was inactivated for 10 min using a boiling water bath and rapidly cooled in an ice bath. Half of the solution was directly centrifuged and freeze-dried, while the remaining was added to 4% trypsin (11,400 U/g) at 37°C for 2 h. After the reaction, enzyme was inactivated for 10 min using a boiling water bath and rapidly cooled in an ice bath. Then the solution was centrifuged and freeze-dried to measure ACE inhibitory activity.

### 2.10. In Silico Simulated Peptide Digestion

This was performed by ExPASy PeptideCutter (accessed on 1 December 2022, https://web.expasy.org/peptide_cutter/). The hydrolysis of identified peptide sequence in MDP was predicted using the known enzymatic cleavage sites. For this study, pepsin, chymotrypsin and trypsin were chosen for the analysis. Eventually, the BIOPEP database was used to identify the ACE–IPs of the hydrolysis fragments derived from digestion.

### 2.11. Statistical Analysis

Every analysis was performed in triplicate. Statistical analysis was performed using SAS 8.1 software. The data were analyzed by one-way analysis of variance (ANOVA), while the significant variation was identified by Duncan’s multiple range test at a 95% confidence level. All the data were presented as mean ±SD. Graphical illustrations were derived by Origin 9.0.

## 3. Results and Discussion

### 3.1. Separation and Purification of MDPH

#### 3.1.1. Ultrafiltration

**Figure 1:**
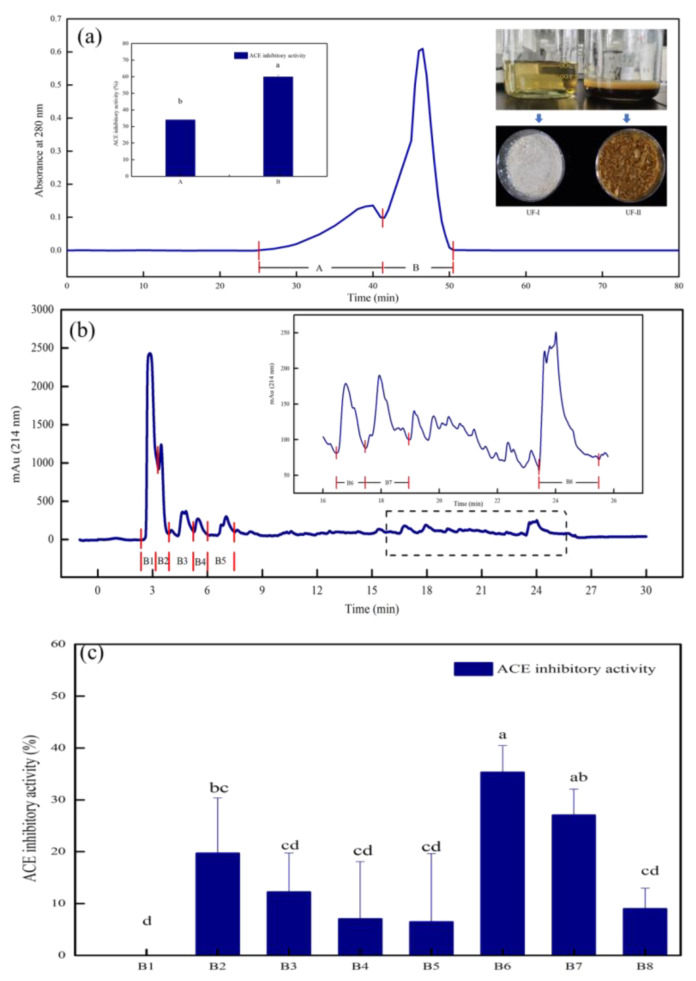
(**a**) Hydrolysates and powders of UF-Ι and UF-II. Sephadex G-25 size exclusion chromatography of peptide and ACE inhibitory activity of fractions obtained by size exclusion chromatography (sample concentration: 60 mg/mL). (**b**) RP–HPLC chromatography of fraction B (sample concentration: 20 mg/mL). (**c**) ACE inhibitory activity of fractions obtained by RP–HPLC (sample concentration: 1 mg/mL). Significant marks in the figure (lowercase letters a–d), containing the same letter means no significant difference (*p* > 0.05).

Ultrafiltration was used to separate, purify and enrich the components of solutions according to the MW of samples [21]. MDP was hydrolyzed by alkaline protease, and the MDPH was separated by a 3 kDa ultrafiltration membrane to produce two components UF-I and UF-II. It can be seen from the Figure 1a that the appearance of UF-Ι and UF-II was significantly different and the ACE inhibitory activity of UF-Ι and UF-II was 66.97 ± 7.40% and 37.08 ± 3.46% at 1 mg/mL, respectively. ACE inhibitory activity was significantly (*p* < 0.05) higher for UF-Ι when compared to UF-II. As expected, ultrafiltration of MDPH led to a decrease in ACE inhibitory activity. The reason may be that UF-II contained more impurities, causing the decrease of ACE inhibitory activity. This result was in agreement with previous research, which indicated that the hydrolysate of <3 kDa from soybean protein isolate exhibited significantly (*p* < 0.05) the highest inhibition of ACE when compared to other fractions, and the ACE inhibitory activity increased with decreasing MW [8].

#### 3.1.2. Gel Filtration Chromatography

To further purification, gel filtration chromatography was used to separate UF-Ι using Sephadex G-25. Gel filtration chromatography was based on MWs to separate peptides or proteins. Chromatograms of UF-Ι are shown in Figure 1a. In this step, UF-Ι was fractionated into two components, named A and B. Compared to component B, the shortest retention time of component A, the largest MW, exhibited significantly (*p* < 0.05) poorest ACE inhibitory activity. MW is one of the most important factors affecting the ACE inhibitory activity of peptides. It is reported that the peptides with strong ACE inhibitory activity had small MW. ACE–IPs usually include 2-12 AAs [22]. Those large MWs are too difficult to bind to ACE active sites, resulting in the decline of ACE inhibitory activity. This result was similar to previous reports showing that the fraction of muscle protein hydrolysate of hard clam (*Meretrix lusoria*) with the smallest MW purified by G-25 gel filtration chromatography presented the highest ACE inhibitory activity [23]. Therefore, component B was selected for the next separation and purification step.

#### 3.1.3. RP–HPLC

RP–HPLC was one of the most common methods for peptide separation, according to the difference in hydrophobic interactions between fractions. Due to its high sensitivity, wide applicability and column efficiency, RP–HPLC was used to purify small molecular samples [24]. Component B, fractionated by ultrafiltration and gel filtration chromatography, was suitable for separation by RP–HPLC. As demonstrated, the sample was fractionated into 8 fractions, namely B1, B2, B3, B4, B5, B6, B7 and B8 (Figure 1b). With the increase of ACN concentration with elution time, hydrophobic peptides were eluted. As seen in Figure 1c, the highest ACE inhibitory activity was obtained in B6. Hydrophobic AAs impact the ACE inhibitory activity of the peptides. Owing to the hydrophobic environment of the ACE C-terminal, the peptides with higher hydrophobic AA content usually expressed higher ACE inhibitory activity.

### 3.2. Identification of ACE–IPs

**Figure 2:**
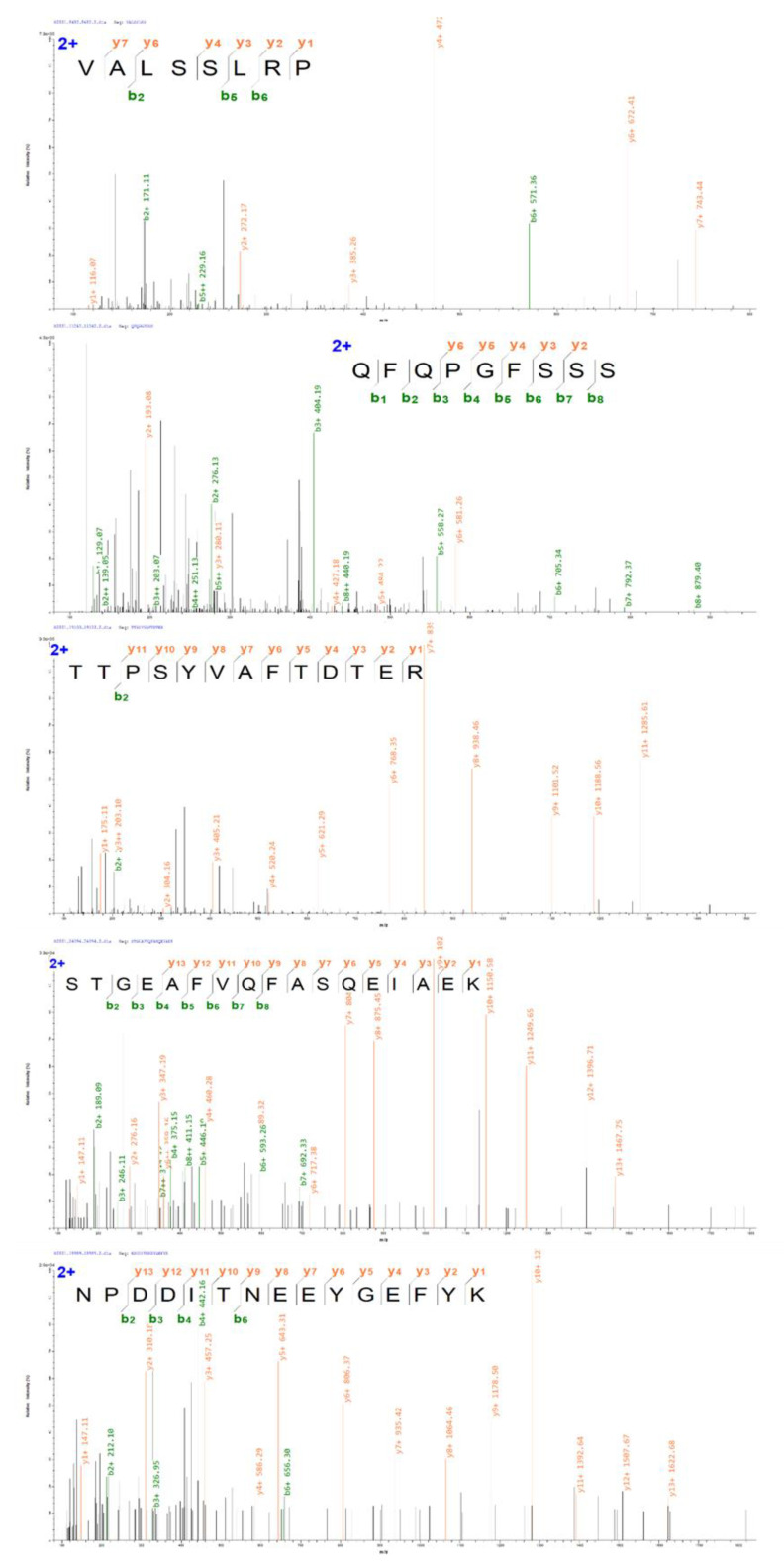
MS/MS spectrums of five ACE–IPs in fraction B6.

To further explore the ACE inhibition mechanism, B6 was selected for identification. The combination of liquid chromatography and MS (LC–MS) has been the most promising method for identifying the AA sequences of peptides because of its good reproducibility, high sensitivity and high efficiency. Electrospray ionization (ESI) is one mass spectrometer instrument with high sensitivity and low detection limit, coupled to LC–MS for identifying peptides [24,25]. In this study, component B6, the most active component after RP–HPLC, was identified by Nano-LC–ESI–MS/MS to obtain the AA sequences potentially responsible for ACE inhibitory activity. A total of 121 peptides were identified. Among all sequences, VALSSLRP represented the highest intensity, which may be integral in the mixture related to ACE inhibitory activity (Table S1). After the AHTPin prediction result was carried out, five peptides with a score above 100 and intensity above 1107 were selected for the synthesis verification test. VALSSLRP, QFQPGFSSS, TTPSYVAFTDTER, STGEAFVQFASQEIAEK and NPDDITNEEYGEFYK were identified from MDP for the first time by enzymatic hydrolysate. The details and MS/MS spectrums of five ACE–IPs are presented (Table 1 and Figure 2).

### 3.3. Validation of ACE Inhibitory Activity

**Figure 3:**
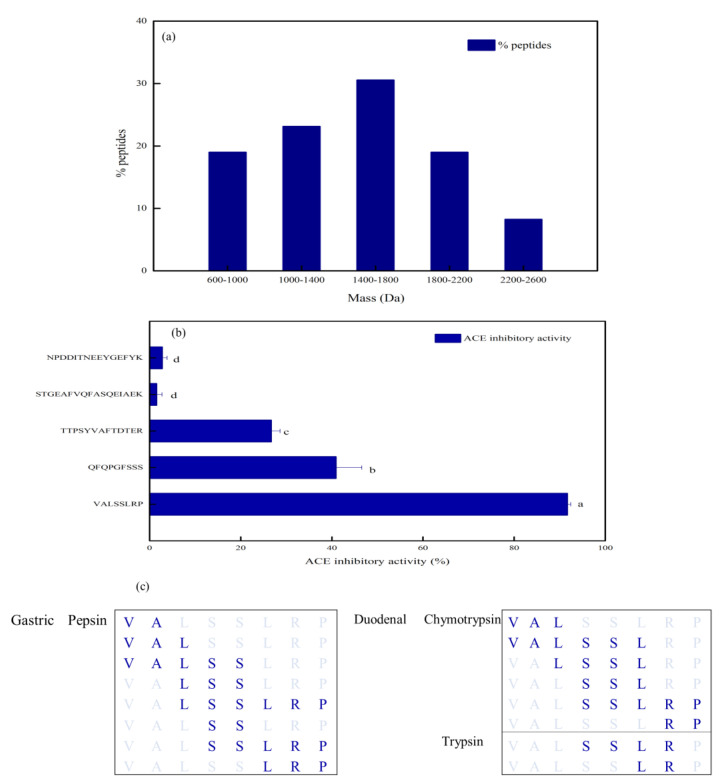
(**a**) Molecular mass distribution of 121 peptides. (**b**) The ACE inhibitory activity of five peptides (sample concentration: 1 mg/mL), significant marks in the figure (lowercase letters a–d), containing the same letter means no significant difference (*p* > 0.05). (**c**) In silico simulated digestion of the peptide VALSSLRP.

The length of the peptides was 8–25, and the mass was between 600 and 2600 Da (Figure 3a). The mass between 1400 and 1800 Da had the highest proportion of 30.58%, while the range of 2200 Da to 2600 Da had the lowest proportion of 8.26%. The range of 600 Da to 1000 Da and 1800 Da to 2200 Da had the same proportion of 19.01%, and the range of 1000 Da to 1400 Da had the proportion of 23.14%. The inhibitory activity of the screened five peptides was determined (Figure 3b). The results showed that VALSSLRP potential containing 8 AA residues was markedly (*p* < 0.05) higher than the other four peptides, reaching 91.67 ± 0.73% at 1 mg/mL (IC50 = 0.039 mg/mL). Nonetheless, the inhibitory activity of the other four peptides was less than 50% at the same concentration. QFQPGFSSS and TTPSYVAFTDTER had significance (*p* < 0.05) in the ACE inhibitory activity of 40.91 ± 5.65% and 26.71 ± 1.91%, respectively. There was no variation (*p* > 0.05), and hardly any ACE inhibitory activity of STGEAFVQFASQUEIAEK and NPDDITNEEYGEFYK with AA sequence lengths of 17 and 15, which were 1.57 ± 1.15% and 2.81 ± 1.03%, respectively.

MW is essential in affecting the inhibitory activity of ACE–IPs. The ACE–IPs usually include 2-12 AA residues [22]. Natesh et al. asserted that the binding effect of the active site of ACE with large MW peptides was ineffective through crystallography [26]. The peptide with long AA sequences is too challenging to enter the ACE active sites, reducing the ACE inhibition effect. In this study, the length of the 5 peptides was between 8–17 AA residues, and the MW was between 800–2000 Da. Among the five peptides synthesized and verified above, the ACE inhibitory activity was inversely proportional to the sequence length of peptides.

Additionally, the hydrophobicity of peptides is another major factor that significantly influences ACE inhibitory activity. The C-terminal domain of ACE is the leading catalytic site of Ang I, which has three catalytic active sites. The hydrophobicity of these catalytic sites is evident, resulting in the formation of the hydrophobic environment of the ACE C-terminal domain [27]. At the same time, Ondetti et al. (1977) [28] proposed that the C-terminal three AA residues of the peptides had the greatest impact on ACE inhibitory activity. When the C-terminal three AA residues were FEP, the peptides had the strongest binding ability with ACE active sites. Therefore, the composition and location of hydrophobic AA residues in ACE–IPs greatly impact the ACE inhibitory effect of peptides [27]. Calculating the proportion of hydrophobic AAs of 5 peptides, it was found that the proportion of VALSSLRP was the highest, accounting for 62.5%, followed by STGEAFVQFASGEIAEK, accounting for 41.18%, and NPDDITNEEYGEFYK was the smallest, accounting for 13.33%. Analyzing the location of hydrophobic AAs in five peptides, the hydrophobic AAs of the VALSSLRP existed in the C-terminal and N-terminal, which was consistent with the characteristics of ACE–IPs. However, STGEAFVQFASGEIAEK had a relatively high proportion of hydrophobic AAs, but the hydrophobic AA residues were located in the middle of the peptide and were difficult to combine with the ACE active pocket, leading to the lowest ACE inhibitory activity. Besides, the long sequence of the peptide hindered the combination of the polypeptide and ACE and affected its activity. Interestingly, similar to lisinopril, enalaprilat and other potent ACE inhibitors (such as captopril, IPP and VPP), VALSSLRP had the same C-terminal Pro residue, which may form strong hydrogen bonds with ACE [29]. In addition, several studies have proved that peptides containing Val, such as VVNE, VVTR, VGVD and VPAAPPK, have strong ACE inhibitory activity [30,31]. In this study, Val may also have contributed to inhibiting ACE activity.

### 3.4. Molecular Docking

Molecular docking is performed to select the best structure of receptor–ligand by calculating the interaction force between receptor and ligand. This optimizes the spatial skeleton of peptides, such as AA side chain, ligand position and bonding angle, to achieve the lowest binding energy [32]. In recent years, ACE–IPs from various food sources have been developed, and their structures have been verified, but the inhibition mechanism of small molecular peptides has not been revealed, which has a specific obstacle to the research and development of ACE inhibitory targeted drugs. The application of molecular docking can break through this bottleneck, clarify the ACE inhibition mechanism and action sites of small molecular peptides and provide some support for the development of targeted drugs.

**Figure 4:**
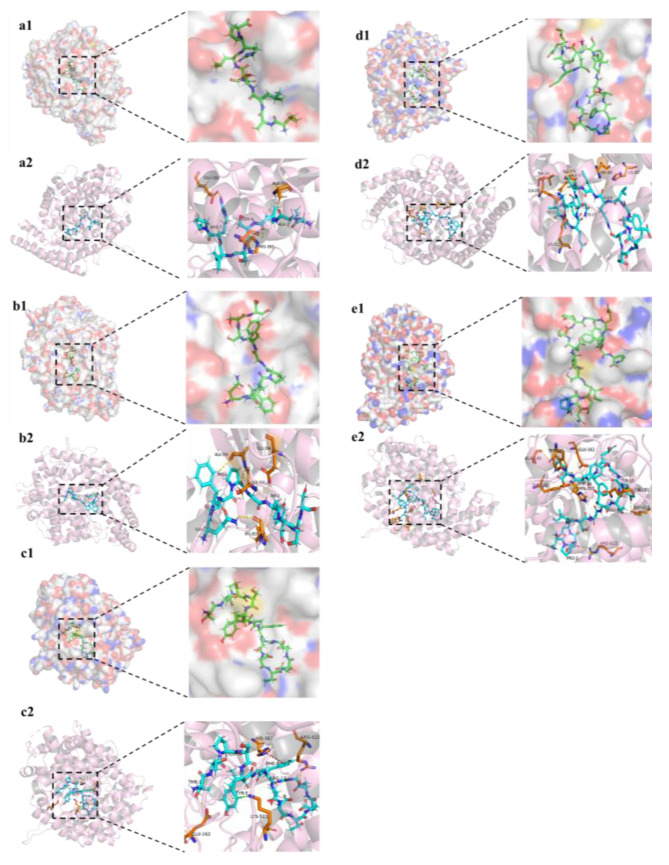
Three-dimensional binding mode of peptide VALSSLRP to ACE (**a1**); Intermolecular interaction between peptide VALSSLRP and ACE (**a2**). Three-dimensional binding mode of peptide QFQPGFSSS to ACE (**b1**); Intermolecular interaction between peptide QFQPGFSSS and ACE (**b2**). Three-dimensional binding mode of peptide TTPSYVAFTDTER to ACE (**c1**); Intermolecular interaction between peptide TTPSYVAFTDTER and ACE (**c2**). Three-dimensional binding mode of peptide STGEAFVQFASQEIAEK to ACE (**d1**); Intermolecular interaction between peptide STGEAFVQFASQEIAEK and ACE (**d2**). Three-dimensional binding mode of peptide NPDDITNEEYGEFYK to ACE (**e1**); Intermolecular interaction between peptide NPDDITNEEYGEFYK and ACE (**e2**). Yellow dash indicates H bonding; red dash indicates π-π interaction; green dash indicates π-cation interaction.

The above result showed that VALSSLRP had strong hydrophobicity and could combine with ACE to inhibit its activity. This study explored the interactions of ACE and peptides using AutoDock Vina 1.1.2, the software to dock the peptide with ACE (PDB ID: 1O86). The docking result was saved according to the conformation of the lowest binding energy, and the interaction mode was presented by Pymol software to analyze the ACE inhibition mechanism. Three-dimensional binding mode and intermolecular interaction of peptide VALSSLRP, QFQPGFSSS, TTPSYVAFTDTER, STGEAFVQFASQEIAEK and NPDDITNEEYGEFYK to ACE were shown in Figure 4. Binding energy is the most intuitive expression of molecular docking results, which can reflect the binding state between receptor and ligand. The lower the binding energy, the structure of the receptor–ligand complex is stable and rational conformational [33]. The binding energy of VALSSLRP, QFQPGFSSS, TTPSYVAFTDTER, STGEAFVQFASQEIAEK and NPDDITNEEYGEFYK were −9.1 kcal/moL, −10.1 kcal/moL, −6.8 kcal/moL, −4.1 kcal/moL and −6.6 kcal/mol, respectively. The result predicted that VALSSLRP and QFQPGFSSS can form more stable structures with ACE than others, and the activity of QFQPGFSSS was higher than VALSSLRP. However, the ACE inhibitory activity of synthesis peptides demonstrating VALSSLRP with the highest activity contradicted the above result. This may be because the molecular docking was predicted with the best structure of receptor–ligand, which was inconsistent with the structure in the actual experimental reaction.

The interactions between ligand and receptor include hydrophobic, van der Waals’s force, hydrogen bond, π bond and electrostatic interaction. Among them, the hydrogen bond interaction may be the strongest [34]. Hydrogen bond is a kind of interaction that is slightly stronger than van der Waals’s force and much weaker than a covalent bond and ionic bond and is vital for the interaction between two molecules. Notably, ACE contains unique active pockets, including one metal ion Zn^2+^ and three active pockets, S1, S2 and S1′, respectively, which are more effective than other interacting sites. Ala354, Glu384 and Tyr523 in S1; Gln281, His353, Lys511, His513 and Tyr520 in S2; and Glu162 in S1′ are key active sites related to inhibiting ACE activity [35]. Similarly, Zn^2+^ can form tetrahedral ligands with ACE AA residues His383, His387 and Glu411, which play a vital role in the stability between receptor and ligand. The docking result showed that VALSSLRP could form three hydrogen bonds with ACE, among which the Arg7 in the peptide chain can form hydrogen bonds with Glu162 in S1′ active pocket, indicating that the peptide can closely bind with S1′ pocket. At the same time, Ala2 and Ser4 in the peptide can form hydrogen bonds with Ala356 and His383 in ACE protein, respectively, strengthening the force between ligand and receptor protein, which may have caused the peptide’s low binding energy. Although some AA residues are not in the ACE active pockets, many studies have shown that these AA residues can form hydrogen bonds with ACE–IPs, which is conducive to the exertion of ACE inhibitory activity. The peptides EKVNELSKD, LHLPLLQ and LQDKIHP, identified from fermented Rubing cheese, can form hydrogen bonds with Ala356 and Arg522, His383, Ser355 and His387 in ACE, respectively, which were not belonging to any active pocket [36]. Zheng et al. (2020) [37] found that SSYYPFK from naked oat globulin hydrolysates could form 11 hydrogen bonds with 8 AA residues of ACE, including Pro407, Agr522, Tyr523, Glu411, His387, Glu384, Asp358 and Ala356, and the residues Glu384 and Tyr523 belong to the ACE active pocket S1.

### 3.5. In Vitro Simulated Pepsin and Trypsin Digestion and In Silico Simulated Peptide Digestion

Compared with before digestion (ACE inhibitory activity was 65.46 ± 1.15%), the ACE inhibitory activity of MDPH decreased significantly (*p* < 0.05) after pepsin treatment for 2 h, which was 42.06 ± 1.79%. After trypsin treatment for 2 h, the ACE inhibitory activity had no significant difference from that before digestion, which was 67.53 ± 1.32% (*p* > 0.05). This may be because the MDPH contained the cleavage site of pepsin. After MDPH had been digested, the active sites with ACE inhibition were reduced, decreasing ACE inhibitory activity. And after trypsin digestion, further hydrolysis led to the exposure of active sites and the enhancement of activity.

The above studies have proved that the MDPH may maintain specific ACE inhibitory activity during digestion. However, it is unclear whether the peptide sequence was easily affected by gastrointestinal digestion to produce new peptide fragments, which may be the reason for the biological activity of MDPH in vivo. Therefore, the changes in peptide fragment formation of VALSSLRP under in silico simulated peptide digestion were studied by PeptideCutter. In silico simulated peptide digestion is to cut the primary structure of peptides through the known cleavage specificity of gastrointestinal enzymes and predict the short peptide sequences that may be released during gastrointestinal digestion. The peptide sequences of VALSSLRP after gastric and duodenal digestion based on in silico simulated peptide digestion were exhibited in Figure 3c. Multiple peptides were obtained after VALSSLRP digestion. Among them, the short peptides LRP and RP have already been reported to be ACE–IPs in the database BIOPEP [38,39]. In addition, LR was a renin inhibitor, which could also reduce blood pressure [40]. Unexpectedly, the peptide VA digested by pepsin was a kind of dipeptidyl peptidase IV inhibitor, regulating the insulin level to achieve adequate blood glucose level [41]. Maybe VALSSLRP also has the function of antidiabetic potential, which can be verified later. Although in silico method is essential for identifying potentially bioactive AA sequences, the results must be carefully interpreted because this procedure does not consider the tertiary protein structure. The inhibition effect of ACE in vivo needs to be verified by in vivo tests in future study.

## 4. Conclusions

This study for the first time isolated and identified the novel ACE–IPs from Muscovy duck blood and explored the interactions between purified peptides and ACE. It was concluded that the component activity of <3 kDa was markedly higher (*p* < 0.05) than that of >3 kDa. The component of <3 kDa was separated by gel filtration chromatography, and two components (UF-I and UF-II) were separated. The ACE inhibitory activity of component B was significantly (*p* < 0.05) higher than that of component A. The component B was separated by RP–HPLC, and eight components were obtained. The activity of component B6 was the highest, and 121 peptide sequences were obtained by Nano-LC–ESI–MS/MS. After screening by AHTPin online website, five peptide sequences were selected for synthesis verification and molecular docking. VALSSLRP, with high ACE inhibitory activity of 91.67 ± 0.73%, can form three hydrogen bonds with ACE and closely combine with ACE protein S1′ pocket in silico simulated peptide digestion. In summary, deriving ACE–IPs from Muscovy duck blood could increase its economic value and promote environmental sustainability. Further study using cell or animal models is necessary to evaluate the ACE inhibitory activity for deeper elucidation.

## Figures and Tables

**Table 1:** Peptides with ACE inhibitory activity in Muscovy duck plasma hydrolysis.

No.	Sequence	Length	Mass/Da	Proteins	Charges	Score	Intensity	Hydrophobicity/%	Hydrophobic Amino Acid Location
1	VALSSLRP	8	841.5022	U3I3Y9	2	111.28	3.29’10^8^	62.50	1,2,3,6,8
2	QFQPGFSSS	9	983.4349	A0A7K7L595	2	113.7	1.37´10^8^	33.33	2,4,6
3	TTPSYVAFTDTER	13	1486.694	U3IT45	2	169.58	9.90´10^7^	30.77	3,6,7,8
4	STGEAFVQFASQEIAEK	17	1840.884	A0A493SXK6	2	136.99	4.10´10^7^	41.18	5,6,7,9,10,14,15
5	NPDDITNEEYGEFYK	15	1832.774	A0A7K7LEE6	2	114.72	1.05´10^7^	13.33	5,13

## Data Availability

The data presented in this study are available in the article.

## Supplementary Materials

The following are available online at https://www.mdpi.com/article/10.3390/foods12010050/s1, Table S1: Peptides identified by LC-MS/MS in the fraction B6 from RP-HPLC.
