# Supplementary material for: ACE Inhibitory Peptides Derived from Muscovy Duck (Cairina moschata) Plasma
Source: Foods. 2022 Dec 22;12(1):50. doi: 10.3390/foods12010050 (PMC9818667; doi:10.3390/foods12010050)
Supplement: Supplementary file 1 [file foods-12-00050-s001.zip › foods-2082396-supplementary material.pdf]

Table S1 Peptides identified by LC-MS/MS in the fraction B6 from RP-HPLC

No.	Sequence	Length	Mass/Da	Proteins	Charges	Score	Intensity
1	AAGIGDLGTGTGPDPAAQWL	20	1866.911	R0KEB6	2	41.052	0
2	AEDNADTLALVFEAPNQEK	19	2073.986	R0K8I7	2	56.648	0
3	AIAELGIYPAVDPLDSTSR	19	1987.026	U3IP65	3	52.784	$75{,}886\times10^{2}$
4	AILVDLEPGTMDSVR	15	1614.829	U3IKI3	2	90.685	$84{,}799\times10^{2}$
5	AIVAIENPADVSVISSR	17	1739.942	U3J7Y9	2	67.385	0
6	ALTVPELTQQVFDAK	15	1658.888	U3IKI3	3	80.632	7.5932×10^{6}
7	ATENDIANFFSPLNPIR	17	1917.959	U3I048	2	81.431	$89{,}869\times10^{2}$
8	ATGEADVEFVTHEDAVAAMSK	21	2176.995	U3I048	3	161.58	7053×10^{4}
9	AVDWWSLG	8	932.4392	A0A493SX27	2	75.311	0
10	AVFPSIVGRPR	11	1197.698	A0A7K7KUC6	3	76.143	$64{,}613\times10^{2}$
11	AVFVDLEPTVIDEVR	15	1700.899	U3IJF6	2	127.87	$92{,}858\times10^{2}$
12	AVLVDLEPGTMDSVR	15	1600.813	A0A7K7KMY6	2	88.441	$16{,}234\times10^{3}$
13	CVRGGDFVFFDGRDSER	17	1960.885	A0A7K7L5A4	2	46.706	0
14	DAGQISGLNVLR	12	1241.673	U3IP36	2	192.91	$94{,}705\times10^{3}$
15	DAGTIAGLNVLR	12	1198.667	U3IT45	2	115.71	$19{,}386\times10^{3}$
16	DLYANTVLSGGTTMYPGIADR	21	2214.063	A0A7K7KUC6	2	47.201	$72{,}135\times10^{2}$
17	DLYEDELVPLFEK	13	1608.792	A0A7K7KZL0	2	79.82	$35{,}543\times10^{2}$
18	DNYVPEVSALDQEIIEVDPDTK	22	2488.186	A0A7K7L561	3	54.223	$74{,}671\times10^{2}$

Table S1. *Cont*

No.	Sequence	Length	Mass/Da	Proteins	Charges	Score	Intensity
19	DYFEEYGKIDTIEIITDR	18	2219.063	A0A7K7L858	3	72.184	$23{,}082\times10^3$
20	EITALAPSTMK	11	1160.611	A0A7K7KUC6	2	112.01	$16{,}503\times10^3$
21	ELAQQVQQVADDYGK	15	1690.816	A0A7K7KS51	2	110.66	$12{,}349\times10^3$
22	EVDEQMLNVQNK	12	1445.682	U3IKI3	2	204.07	0
23	FEAGHDAF	8	892.3715	U3IU92	2	104.11	$19{,}767\times10^3$
24	FESPEVAER	9	1062.498	A0A493T8B8	2	66.692	0
25	FLAYPQTK	8	966.5175	P04442	2	154.35	122×10^6
26	FQPGFSSS	8	855.3763	A0A7K7L595	1	117.18	223×10^6
27	FSGSKSSSTAMLT	13	1302.613	A0A493TNK9	2	83.948	$10{,}493\times10^3$
28	FVGDNYYRVN	10	1245.578	A0A7K7KQP1	2	147.29	$15{,}838\times10^3$
29	GAEAANVTGPGGVPVQGSK	19	1694.859	A0A7K7KW68	2	78.814	0
30	GEATVSFDDPPSAK	14	1419.652	A0A7K7LGU0	2	102.4	0
31	GFGFVTFDDHDPVDKIVLQK	20	2276.148	A0A7K7L858	4	59.512	$51{,}425\times10^2$
32	GHQPGVTF	8	841.4083	U3IF27	2	111.69	$28{,}437\times10^3$
33	GKDAIAQF	8	848.4392	A0A7K7L9Q3	2	87.754	196×10^6
34	GSYGDLGGPIITTQVTIPK	19	1916.026	U3IU09	2	61.375	$13{,}648\times10^3$
35	GVAADYEHAL	10	1044.488	U3IU92	2	66.682	0
36	GVEEEEEDGEMRE	13	1536.589	A0A7K7KK46	2	90.685	$10{,}023\times10^3$

Table S1. *Cont*

No.	Sequence	Length	Mass/Da	Proteins	Charges	Score	Intensity
37	GVPQIEVTFDIDANGIVHVSAK	22	2308.206	U3IP36	3	45.784	15,705×10^3
38	HISGPGSF	8	800.3817	A0A7K7L595	2	88.201	0
39	HTGPNSPDTANDGFVR	16	1683.76	A0A493SXK6	3	91.657	0
40	IAPPEAPVTGYMFGK	15	1576.796	U3J3C9	2	77.64	48,638×10^2
41	IDTIEIITDR	10	1187.64	A0A7K7L858	2	138.4	18,724×10^3
42	IFEAGRDV	8	905.4607	A0A7K7LC88	2	62.714	0
43	IFVGGLNPEATEEK	14	1502.762	R0JDB7	2	168.3	69,815×10^3
44	IFVGGLSPDTPEEK	14	1487.751	A0A7K7LE07	2	179.57	92,035×10^3
45	IIDVVYNASNNELVR	15	1717.9	R0L3S3	2;3	99.215	60,292×10^2
46	IINEPTAAAIAYGLDK	16	1658.888	U3I640	2;3	100.55	1716×10^4
47	IITITGTQDQIQNAQYLLQNSVK	23	2588.381	U3IU09	3	87.156	45,867×10^2
48	INATNIDSFKNCTK	14	1567.766	A0A493TAW2	2	99.568	54,516×10^3
49	IPPSFSPLVL	10	1068.622	U3IYP8	2	70.268	0
50	IREYFGEFGEIEAIELPMDPK	21	2482.209	R0JDB7	3	55.588	50,171×10^2
51	ISVYYNEATGGK	12	1300.63	U3IKI3	2	93.096	95,377×10^2
52	ITITNDQNR	9	1073.547	U3I640	2	116.73	46,523×10^3
53	ITPSYVAFTPEGER	14	1565.773	U3I640	2	97.734	14,686×10^3
54	KSQVFSTAADGQTQVEIK	18	1935.99	U3IP36	3	85.808	88,807×10^2

Table S1. *Cont*

No.	Sequence	Length	Mass/Da	Proteins	Charges	Score	Intensity
55	LAPDYDALDVANK	13	1403.693	U3HYT9	2	104.52	1439×10^4
56	LIEVDDER	8	987.4873	R0K7Y6	2	70.919	1733×10^4
57	LKGEATVSFDDPPSAK	16	1660.831	A0A7K7LGU0	2	68.277	14,789×10^3
58	LNSSTFLN	8	894.4447	A0A7K7KT01	2	71.342	14,116×10^3
59	LPESYAQSIYMQRRN	15	1854.905	A0A7K7LKZ6	2	56.563	0
60	LPSTSPVTGY	10	1020.513	U3IZ83	2	65.038	0
61	LSSPPSVNFV	10	1045.544	A0A493TVU4	2	67.169	0
62	LTPEEIER	8	985.508	U3I640	2	62.546	0
63	LVAIVDVIDQNR	12	1353.762	U3I808	2	85.377	13,884×10^2
64	LVEAAAKN	8	814.4549	R0KCT6	2	60.788	0
65	LVQDVANNTNEEAGDGTTTATVLAR	25	2559.241	A0A7K7LE25	3	66.3	0
66	MIETAQVDER	10	1190.56	R0JL85	2	76.827	0
67	NAVITVPAYFNDSQR	15	1693.842	U3IP36	2	148.18	63,563×10^3
68	NFILDQTNVSAAAQR	15	1646.838	R0L9Z5	2;3	213.16	24,578×10^3
69	NIQGAQDMGVPSAWV	15	1571.74	R0JVJ2	2	58.699	0
70	NLMLDRLS	8	960.5063	R0LI37	2	71.296	0
71	NMGGPYGGGNYGPGGSGGSGGYGGR	25	2188.898	A0A7K7L858	2	149.09	1322×10^4
72	NPDDITNEEYGEFYK	15	1832.774	A0A7K7LEE6	2	114.72	10,459×10^3

Table S1. *Cont*

No.	Sequence	Length	Mass/Da	Proteins	Charges	Score	Intensity
73	NQLTSNPENTVFDAK	15	1676.801	U3I640	2	147.71	$24,117 \times 10^3$
74	NQVAMNPTNTVFDAK	15	1648.788	U3IT45	2	83.617	0
75	NVQAEEMVEFSSGLK	15	1666.787	A0A7K7LHY3	2	117.23	0
76	PISETEPQEDKGSQINV	17	1869.896	R0JIU5	2	61.353	$92,384 \times 10^2$
77	QFQPGFSSS	9	983.4349	A0A7K7L595	2	113.7	137×10^6
78	QFQPGFSSSG	10	1040.456	A0A7K7L595	2	102	0
79	QQLSAEELDAQLDAYNAR	18	2033.965	R0KBD0	3	67.449	$42,623 \times 10^3$
80	RVPELMDSQ	9	1073.518	A0A7K7LNG1	2	113.71	$18,454 \times 10^3$
81	SDGAITWNN	9	976.425	A0A7K7LNG1	1	103.56	$25,521 \times 10^3$
82	SDIGEVILVGGMTR	14	1445.755	U3IP36	2	108.35	$16,139 \times 10^3$
83	SEAEELPGMELRGLQLGYPQ	20	2216.078	A0A7K7KQ17	2	41.54	0
84	SELSNLHAY	9	1032.488	P04442	2	81.297	0
85	SGNFGGSR	8	780.3515	A0A7K7L858	2	91.853	0
86	SLAGDLEK	8	831.4338	A0A493T6Z7	2	62.717	0
87	SLLDSRLRAANE	12	1343.716	R0K202	2	88.948	$20,263 \times 10^3$
88	SLPSNVVQDSGRAY	14	1491.732	U3I742	2	79.741	$43,697 \times 10^2$
89	SPQLRDQI	8	955.5087	A0A493TKR2	2	68.915	0
90	SPSTVFNHY	9	1050.477	A0A7K7KQP1	2	176.41	$22,777 \times 10^3$

Table S1. *Cont*

No.	Sequence	Length	Mass/Da	Proteins	Charges	Score	Intensity
91	SQIFSTASDNQPTVTIK	17	1835.927	U3I640	2;3	139.3	48,176×10^3
92	SQVFSTAADGQTQVEIK	17	1807.895	U3IP36	2;3	226.23	85,655×10^3
93	SRGFGFVTFSSMAEVDAAMAAR	22	2307.078	A0A7K7L858	3	45.363	0
94	SSGSPYGGGYGSGSGSGGYGGR	22	1909.783	R0LJ35	2	65.472	32,667×10^3
95	STESLQANVQR	11	1231.616	R0LLL2	2	163.72	13,027×10^3
96	STGEAFVQFASQEIAEK	17	1840.884	A0A493SXK6	2	136.99	40,989×10^3
97	SVGDGETVEFDVVEGEK	17	1794.816	A0A7K7KW68	2	79.633	36,812×10^2
98	SVTEQGAELSNEER	14	1547.706	R0L3T4	2	79.693	52,439×10^2
99	SYELPDGQVITIGNER	16	1789.885	A0A7K7KUC6	2	72.089	24,625×10^3
100	TDASSASSFLDSDELER	17	1828.796	R0LPV2	2	48.659	0
101	TDYNASVSVPDSSGPER	17	1779.791	U3IU09	2	130.15	35,011×10^3
102	TGYTLDVTTGQR	12	1310.647	A0A7K7KZL0	2	146.11	1403×10^4
103	TIGGGDDSFNTFFSETGAGK	20	2006.886	U3IJF6	2;3	65.805	2923×10^4
104	TITLEVEPSDTIENVK	16	1786.92	Q7ZSY5	2;3	160.15	39,164×10^3
105	TSIAIDTIINQK	12	1315.735	A0A7K7LHY3	2	85.29	47,152×10^2
106	TTPSVVAFTADGER	14	1449.71	U3IP36	2;3	130.01	135×10^6
107	TTPSYVAFTDTER	13	1486.694	U3IT45	2	169.58	98,999×10^3
108	TVIDTSGG	8	748.3603	R0LWA9	1	118.69	60,957×10^2

Table S1. *Cont*

No.	Sequence	Length	Mass/Da	Proteins	Charges	Score	Intensity
109	TVNELQNLTAAEVVVPR	17	1852.005	R0L8J7	2;3	113.76	$22{,}749\times10^{3}$
110	VALSSLRP	8	841.5022	U3I3Y9	2	111.28	329×10^{6}
111	VAPEEHPVLLTEAPLNPK	18	1953.057	A0A7K7KUC6	3	63.145	$85{,}334\times10^{3}$
112	VEIIANDQGNR	11	1227.621	U3I640	2	153.05	$26{,}998\times10^{3}$
113	VINEPTAAALAYGLDK	16	1644.872	U3IP36	2	100.55	$48{,}684\times10^{3}$
114	VINEPTAAALAYGLDKSEDK	20	2104.069	U3IP36	3	85.231	7392×10^{3}
115	VISSIEQK	8	902.5073	R0JH47	2	117.16	$73{,}211\times10^{2}$
116	VTDALNATR	9	959.5036	A0A7K7LE25	2	156.51	$76{,}062\times10^{2}$
117	VTGEADVEFATHEDAVAAMSK	21	2176.995	A0A493SXK6	3	63.184	$10{,}188\times10^{3}$
118	VVDLLAPYAK	10	1087.628	U3IP65	2	68.224	0
119	WDAPAVTVR	9	1013.529	U3IZ83	2	113.26	$26{,}526\times10^{3}$
120	WITSIGLQ	8	916.5018	U3I182	2	62.715	0
121	WKEELQDL	8	1059.524	A0A493TLU3	2	86.086	0
